# A Quantitative Proteomic Analysis of Brassinosteroid-induced Protein Phosphorylation in Rice (*Oryza sativa* L.)

**DOI:** 10.3389/fpls.2017.00514

**Published:** 2017-04-07

**Authors:** Yuxuan Hou, Jiehua Qiu, Yifeng Wang, Zhiyong Li, Juan Zhao, Xiaohong Tong, Haiyan Lin, Jian Zhang

**Affiliations:** ^1^State Key Lab of Rice Biology, China National Rice Research InstituteHangzhou, China; ^2^Agricultural Genomes Institute at Shenzhen, Chinese Academy of Agricultural SciencesShenzhen, China

**Keywords:** rice (*Oryza sativa* L.), brassinosteroid, signaling, phosphorylation, proteome

## Abstract

The group of polyhydroxysteroid phytohormones referred to as the brassinosteroids (BRs) is known to act on plant development and the stress response. BR signal transduction relies largely on protein phosphorylation. By employing a label-free, MS (Mass Spectrometry)-based phosphoproteomic approach, we report here the largest profiling of 4,034 phosphosites on 1,900 phosphoproteins from rice young seedlings and their dynamic response to BR. 1,821 proteins, including kinases, transcription factors and core components of BR and other hormone signaling pathways, were found to be differentially phosphorylated during the BR treatment. A Western blot analysis verified the differential phosphorylation of five of these proteins, implying that the MS-based phosphoproteomic data were robust. It is proposed that the dephosphorylation of gibberellin (GA) signaling components could represent an important mechanism for the BR-regulated antagonism to GA, and that BR influences the plant architecture of rice by regulating cellulose synthesis *via* phosphorylation.

## Introduction

In addition to the five well recognized phytohormones (the auxins, cytokinins, gibberellins (GA), abscisic acid (ABA) and ethylene), brassinosteroids (BRs) are a class of polyhydroxysteroids that have been recognized as the sixth class of plant hormones. Over 70 BR compounds have been identified from plants (Bajguz, 2007), where they contribute to cell elongation and proliferation, leaf senescence, vascular differentiation, plant architecture, flowering time and germination (Wang et al., 2012; Guo et al., 2013; Wang W. et al., 2014; Zhang C. et al., 2014). They work synergistically with auxin, but also engage in cross-talk with other hormones such as GA, ABA (Gallego-Bartolome et al., 2012), and ethylene (Guo et al., 2013).

The details of BR signaling model in *Arabidopsis thaliana* have been well elucidated (Wang et al., 2012; Wang W. et al., 2014). In this model, BR are sensed by the extracellular domains of the membrane receptor BRI1 (BRASSINOSTEROID INSENSITIVE 1) and BAK1 (BRI1 Kinase Insensitive 1) (Santiago et al., 2013; Sun et al., 2013). In the presence of BRs, BRI1 directly binds with BRs to form a special crystal structure favoring the binding of the co-receptor BAK1. BRI1 is activated within the BRI1-BR-BAK1 complex *via* the autophosphorylation and BAK1-mediated transphosphorylation of its kinase domain, which enables it to phosphorylate both BSK1 (Brassinosteroid-Signaling Kinase 1) and CDG1 (Constitutive Differential Growth 1) (Oh et al., 2015). The latter phosphorylates BSU1 (BRI1 Suppressor 1), a phosphatase which acts to suppress the kinase activity of BIN2 (Brassinosteroid Insensitive 2) *via* its dephosphorylation (Mora-Garcia et al., 2004; Tang et al., 2008b; Kim et al., 2009). Upon the BIN2 inactivation, BZR1 (Brassinazole Resistant 1) and BZR2 (Brassinazole Resistant 2, also named as BES1) are dephosphorylated by PP2As (Phosphatase 2A), and are accumulated in nucleus, where they regulate the expression of BR responsive genes. In the absence of BRs, activated BIN2 phosphorylates BZR1 and BZR2 to inhibit their nuclear localization and DNA-binding activity, and ultimately blocks the BR signaling pathway (He et al., 2002; Wang et al., 2002; Yin et al., 2002). Almost in all the known BRs-related signaling pathways, signal is transduced in forms of phosphate. It is therefore that exploring the protein phosphorylation, in particular the phosphorylation sites, intensities and dynamics in BRs signaling has attracted great attentions. A number of critical residues in both BRI1 and BAK1 have been identified. A particularly critical site is the residues lying within the BRI1 and BAK1 activation loops, although both Thr-1180 and Ser-1162, which lie outside of the activation loop, are also stimulatory with respect to kinase activity (Oh et al., 2000; Wang et al., 2005, 2008). A substitution of Ser-891 into Thr-891 remains but delays the catalyzing activity of BRI1. Interestingly, phosphorylation may impose opposite effects on the function of different proteins. For example, phosphorylation of Tyr-610 on BAK1 is essential for BR signaling *in vivo* (Oh et al., 2010), while autophosphorylation on Tyr-831, Tyr-956, and Ser-891 of BRI1 could inhibit the receptor kinase activity and terminate the BR signaling (Oh et al., 2009, 2012). In addition, BRI1 phosphorylates BKI1 (BRI1 Kinase Inhibitor 1) on Tyr-211 in the transphosphorylation, leading to the disassociation of BKI1 from the plasma membrane (Wang et al., 2011). Nevertheless, the overall level of understanding of protein phosphorylation in the context of BR signaling remains patchy, mainly because conventional assays can only focus on a single kinase-substrate pair. On the other hand, protein phosphorylation is highly dynamic to fine-tune the BR signaling under different physiological conditions, thus the phosphorylation intensity, in addition to the phosphorylation status, are also key to understand the BR signaling process. To achieve this, quantitation of protein phosphorylation by quantitative phosphoproteomic methods is certainly important. Previous attempts to identify BR-induced protein phosphorylation by coupling gel electrophoresis with liquid chromatography/mass spectrometry (MS) have at best identified only a small number of phosphoproteins in Arabidopsis (Deng et al., 2007; Tang et al., 2008a; Shigeta et al., 2011). Until recently, novel phosphopeptide enrichment methods, advanced MS, and sophisticated algorithms have promoted the process of wide-scale phosphoprotein identification, especially in a quantitative manner. Lin et al. (2015) profiled 1104 phosphopeptides of 739 unique phosphoproteins induced by BR in *A. thaliana*. This research constructed a time-dependent kinase-substrate interaction network, and revealed complicated cross-talk between BRs with other phytohormone signaling such as auxin and ABA (Lin et al., 2015).

Rice (*Oryza sativa* L.) is one of the most important crops in the world, providing calories for over half of the global population. Meanwhile, rice is also a model plant for molecular biology research due to its small genome size, released reference sequence, ample genetic resources and co-linearity with other grasses (Zhang et al., 2007). In rice, the majorities of mutants which are either BR-deficient or BR-insensitive are dwarfed in stature and tiller profusely (Hong et al., 2003; Tanabe et al., 2005; Sakamoto et al., 2013). By controlling the leaf angle and thereby increasing the leaf area index of the plant, BRs make a positive contribution to the plant’s productivity (Sinclair and Sheehy, 1999; Sakamoto et al., 2012). Recently, BRs is also implicated in grain size determination and stress response in rice (Zhang C. et al., 2014). Unlike the well-documented BR signaling pathway in Arabidopsis, only a rudimentary understanding of BR signaling is currently available in rice. Homologs of several critical *A. thaliana* genes, notably *BRI1*, *BAK1*, *GSK1* and *BZR1*, have been shown to encode rice proteins which act in the same way as they do in *A. thaliana*, while others are not represented in rice and still others are not functionally related. The indication is therefore that certain aspects of BR signaling are not conserved between rice and *A. thaliana*. The present research set out to obtain a clearer picture of BR-induced phosphorylation in rice, by initiating a quantitative phosphoproteomic analysis of seedlings exposed to exogenous BR. The experiment has revealed a substantial set of phosphosites and phosphoproteins, while also exposing aspects of BR signaling in this important crop and model species.

## Materials and Methods

### Plant Materials and BR Treatment

The Nipponbare (*Oryza sativa* L. ssp *japonica*) plants used in this study were grown by hydroponics method in growth chambers (90% relative humidity, 30/28°C, 14 h light/10 h dark cycle). Two-week old young seedlings with hydroponics were soaked into water containing 10 μM epibrassinolide (Cat No. E1641, Sigma, St. Louis, MN, USA) for 24 hours, during which samples were collected at different time points (0 h, 3 h, 6 h, 12 h, and 24 h) and immediately stored in liquid nitrogen until use. Three biological replicates were performed for each treatment and the control.

### Quantitative RT-PCR

Total RNA of the samples after BR treatment was isolated using Trizol (Invitrogen, Carlsbad, CA, USA) according to the manufacturer’s manual. Four micrograms of total RNA was performed for reverse transcription using first strand cDNA synthesis Kit (Toyobo, Shanghai, China). The primer pairs were set to detect the expression level of *ILI1* (*LOC_Os04g54900*) (forward: 5′ ATGTCGAGCAGCCGGAGGTC 3′, reverse: 5′ CGTCTCGCTGAGGTTGTCC 3′), *BUI1* (*LOC_Os06g12210*) (forward: 5′ CGACGACGAAGCTGCTGA 3′, reverse: 5′ CGCCTGGGCTGTTGTGAT 3′), *IBH1* (*LOC_Os04g56500*) (forward 5′ CCGCCGAACCCTAACCCTAG, reverse 5′ CAGGAAGTGGAAGGCCAGCAT) and ubiquitin gene was used as an internal control (primer forward 5′ CACCCTGGCTGACTACAACA 3′, reverse 5′ TTCTTCTTGCGGCAGTTGAC 3′). Real-time quantitative RT-PCR was performed in a total reaction volume of 20 ml (10 μL THUNDERBIRD SYBR^®^ qPCR Mix (Toyobo, Shanghai, China), 1 μL cDNA, 1 μL primers, and 8 μL water) on the Bio-Rad CFX96 real-time PCR detection system (Bio-Rad, Hercules, CA, USA). The relative expression level was calculated by the 2^-ΔΔCT^ method. The experiment was performed in three biological replicates.

### Phosphopeptide Preparation

The total protein extraction and digestion were performed by exactly following the methods described by (Hou et al., 2015). For phosphopeptides enrichment, 1 mg digested peptides were dissolved with binding buffer (80% ACN, 5% TFA, 1 M lactic acid), then incubated with 4 mg TiO_2_ beads (GL sciences, Torrance, CA, USA) for three times, each time for 30 minutes at room temperature. The totally 12 mg beads were then washed with 500 μL binding buffer for twice and 500 μL washing buffer (80% CAN, 0.5% TFA) for twice. All TiO_2_ beads were transferred into a 200 μL homemade StageTip containing two layers of C18 solid phase extraction disk (3M, St. Paul, MN, USA). The StageTip was centrifuged at 300 *g* for 10 min to discard the flow through. The enriched phosphopeptides were incubated with 150 μL elution buffer (40% ACN, 15% NH_3_H_2_O) for four times. The combined 600 μL eluates were subsequently dried to ∼5 μL in a SpeedVac and reconstituted with 5% MeOH in 1% TFA solution for LC-MS/MS analysis.

### LC-MS/MS and Data Analysis

This was done in Beijing Proteome Research Center. LC-MS/MS analyses were performed on an Easy-nLC 1000 liquid chromatography system (Thermo, Waltham, MA, USA) coupled to a Q-Exactive Plus *via* a nano-electrospray ion source (Thermo, Waltham, MA, USA). The peptide mixture was eluted from a 360-μm ID × 2 cm, C18 trap column and separated on a homemade 100 μm ID × 10 cm column with a linear 5–35% acetonitrile gradient at 500 nl/min. Survey scan were acquired after accumulation of 3e^6^ ions in Orbitrap for m/z 300–1400 using a resolution of 70,000 at m/z 400. The top 20 most intense precursor ions were selected for fragmentation in the HCD cell at normalized collision energy of 27%, and then fragment ions were transferred into the Orbitrap analyzer operating at a resolution of 17,500 at m/z 400. For the phosphopeptide identification and phosphosite quantification, raw spectral data were processed in Proteome Discoverer 1.4.1.14 suites with Mascot search engine against the rice genome annotation project database^1^. The mass tolerance was set at 20 ppm for precursor, and 50 mmu for the tolerance of product ions. Oxidation (M), Acetyl (Protein-N term), and Phospho(S/T/Y) were set as variable modifications, and Carbamidomethyl (C) as static modification in the Mascot searches for phosphopeptides. Two missed cleavage on trypsin was allowed. The results were filtered for peptide with False discovery rates <1% by the Percolator tool of the Protein Discoverer package. PhosphoRS software within the Protein Discoverer software suite were used for an automatically re-analyzing of all the phosphopeptide hits, and those phosphorylation sites with a PhosphoRS probability higher than 90% were accepted as localized. Only those peptides which were phosphorylated in at least two of the three biological replicates were considered as truly phosphorylated. The phosphorylation is quantified based on the peak area under the ion intensity by using precursor ions area detector in PD1.4.1.14. Within-group means were calculated to determine fold changes. The differentially phosphorylated (DP) protein was defined to have over twofold changes in the average intensity with credible student’s *t*-test (*P* < 0.05).

### Western Blot Analysis

CIAP treatment was performed by adding 1 μL of calf intestinal alkaline phosphatase (Takara, Dalian, China) into 20 μg protein of each sample for 30 min at 37°C. Then, Western blot was conducted following (Hou et al., 2015). The prepared protein samples were resolved in 10% SDS-polyacrylamide gel, and transferred onto a 0.45 μm polyvinylidene fluoride fluoropolymer (PVDF) membrane (Millipore, Darmstadt, Germany) by using an electrophoretic blotting system (Bio-Rad, Hercules, CA, USA). The immune-blot were detected with corresponding primary antibodies (1:1000 dilution), secondary antibody IgG conjugated with HRP (1:20,000 dilution), and visualized by using the enhanced chemiluminescence (Pierce, Waltham, MA, USA). β-tubulin protein was used as the internal control. The band intensities were quantified using the ImageJ software according to the instructions^2^. All the sample intensities were first normalized to the control β-tubulin, and then calculated based on the ratio to set the relative level of 0 h into 1. The primary antibody against OsBZR1, D1, GID2, SAPK9, and SMG1 were commercially synthesized by immunizing rabbits and affinity purified by GenScript Company (Nanjing, China). The antigenic determinant peptide sequences are D1/RGA1: CSRSHSLSEAETTK; SMG1: MRPGGPPSLRAGLQC; SAPK9: MERAAAGPLGMEMPC; GID2: MSQPAELSREENVYC, and BZR1: CRPPKIRKPDWDVDP. Anti-β-tubulin (Cat No. M20005) were purchased from Abmart Company (Shanghai, China).

## Results

### Identification of Phosphorylation Sites, Peptides, and Proteins

**FIGURE 1:**
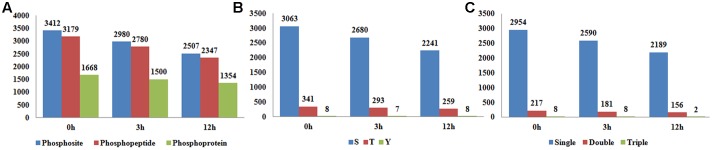
**(A)** The number of identified phosphosites, phosphopeptides, and phosphoproteins in samples at 0, 3, and 12 h after brassinosteroid (BR) treatment. **(B)** The counts of phosphosites in serine, threonine and tyrosine. **(C)** The counts of phosphopeptides carrying single, double, and triple phosphorylation modifications.

Phytohormone signaling is majorly transferred *via* protein phosphorylation cascades (Walton et al., 2015). In an effort to explore the roles of protein phosphorylation in BR signaling, we profiled the phosphoproteome of Nipponbare (*Oryza sativa* L. *ssp japonica*) 14 DAG (Day After Germination) seedlings by using a quantitative, label-free phosphoproteomic approach. Given that obvious genomic response to BR started at 3 h after treatment and robust physiological responses were observed after 12 h (Goda et al., 2002; Deng et al., 2007), three time points 0 hour (0 h), 3 hour (3 h), and 12 hour (12 h) after BR treatment, which represent the control, early BR response and late BR response, respectively, were selected for the proteome assay. Prior to the proteomic assay, we checked the mRNA expression level of three BR responsive genes, including two BR-induced genes *ILI1* (*Increased Leaf Inclination 1*) (*LOC_Os04g54900*) and *BU1* (*Brassinosteroid Upregulated 1*) (*LOC_Os06g12210*) and one BR-repressed gene *IBH1* (*ILI1 binding bHLH*)(*LOC_Os04g56500*), at five time points (Tanaka et al., 2009; Zhang et al., 2009). As indicated in the qRT-PCR result, the expression levels of the three genes were vigorously altered, confirming a valid BR treatment on the plants in this experiment (**Supplementary Figures S1A**–**C**). The LC-MS/MS assay identified 3,412 phosphosites on 3,179 phosphopeptides at 0 h, 2,980 phosphosites on 2,780 phosphopeptides at 3 h and 2,507 phosphosites on 2,347 phosphopeptides at 12 h (**Figure 1A**). Of the phosphosites detected at 0 h, 89.7% were phosphoserine, 9.9% were phosphothreonine, and 0.4% were phosphotyrosines (**Figure 1B**), and the proportions were similar at the other two sampling times. Emerging evidences have shown that the distribution ratios of phosphorylation amino acids are much conserved in plants, despite the great variety of species, tissue and treatment applied (Han et al., 2014; Lv et al., 2014; Wang K. et al., 2014; Zhang M. et al., 2014; Hou et al., 2015; Qiu et al., 2016). In the three samples, 92.9–93.3% of the peptides carried only one phosphorylation group, 6.5–6.8% of the peptides carried two phosphorylation modifications, whereas only less than 0.3% of peptides had more than two phosphorylation modifications (**Figure 1C**). The 0, 3, and 12 h phosphopeptides were corresponded to 1668, 1500, and 1354 phosphoproteins, respectively, which represented the largest scale identification of BR responsive phosphoproteins in plants thus far.

### Conserved Sequence Motifs and Structures Flanking the Phosphosites

**FIGURE 2:**
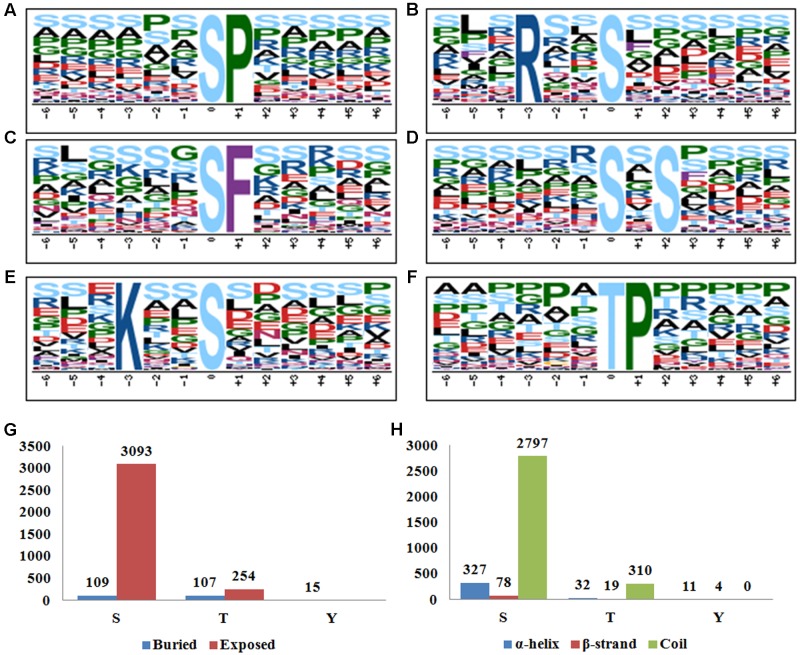
**(A–F)** Motif-X analysis of the significantly enriched phosphorylation motifs around the phosphosites of the differentially phosphorylated (DP) proteins in response to BR treatment. **(A)** [sP], **(B)** [Rxxs], **(C)** [sF], **(D)** [sxS], **(E)** [Kxxs], **(F)** [tP]. **(G)** Surface accessibility of the identified phosphosites by using NetsurfP. **(H)** Secondary structure analysis of the sequences flanking phosphosites.

As revealed by previous mega data analysis, featured motifs around the phosphosites provided valuable information of the kinase-substrate specificities. In this study, Motif-X^3^ (Chou and Schwartz, 2011) was employed to search the enriched motifs around phosphosites identified. The full set of 3,659 distinct phosphosites (3,283 phosphoserines, 361 phosphothreonines, and 15 phosphotyrosines) revealed at all three sampling times was submitted for analysis by motif-X software. As shown in **Figure 2**, at least five phosphoserine motifs (*n* > 200) and one phosphothreonine motif (*n* > 200) were identified (**Figures 2A–E**). As reported by many studies, [sP], [sxS], and [Rxxs] were frequently recurring (respectively, 1,498, 955, and 830 times) motifs, while the motif [Kxxs] (a basic S type) was recorded 266 times. [sF] was annotated to be a low frequency motif in plants by a previous meta-analysis (van Wijk et al., 2014). However, it seemed to be not the case, at least in rice, because two recent studies both found that [sF] was over-represented in rice leaves and developing seeds (Hou et al., 2015; Qiu et al., 2016). In the current study, we detected 252 hits of [sF] in rice young seedlings, which again supported that [sF] is a highly conserved motif for phosphorylation. [tP] was the only motif associated with phosphothreonine here (**Figure 2F**), while we did not found any conserved motifs for phosphotyrosine possibly due to the limited number of input sequences.

NetsurfP^4^ has been routinely employed to predict the surface accessibility and potential conserved secondary structures based on primary amino acid sequences (Petersen et al., 2009). By using the Netsurfp online tool, we found that 3093 of the 3283 (94.2%) phosphoserines and 254 of the 361 (70.4%) phosphothreonines lay on an exposed surface of the proteins, which is in agreement with the assumption that a surface location facilitates the access for the phosphorylation catalyzing enzyme(s) (**Figure 2G**). Nevertheless, all of the 15 phosphotyrosines were likely buried within the protein, displaying a divergent pattern from the other two phosphosite types. For the protein secondary structures, 327 (10.0%), 78 (2.4%), and 2797 (77.6%) of the phosphoserines were located in α-helix, β-strand and coil structures, respectively (**Figure 2H**). A similar distribution obtained for the phosphothreonines, implying a preference for coils with respect to the phosphorylation of both serine and threonine. The pattern for the phosphotyrosines was rather different: 73.3 and 26.7% of the locations were present in, respectively, an α-helix and a β-strand, with none present in a coil structure. The divergent results in surface accessibility and secondary structures implied different mechanisms of phosphorylation between serine/threonine and tyrosine.

### DP Proteins in Response to BR Treatment

**FIGURE 3:**
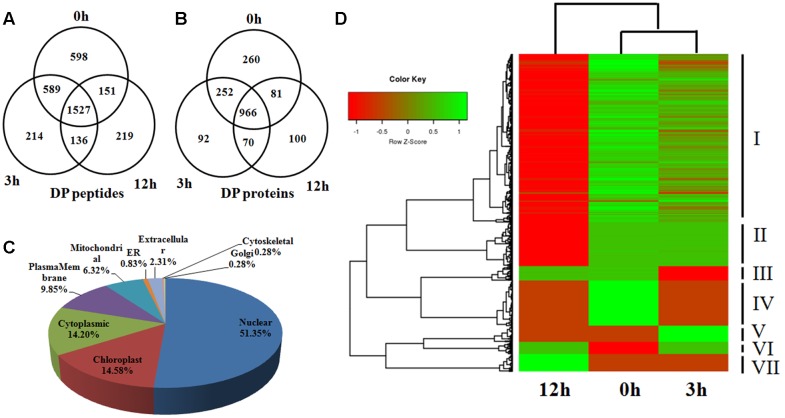
**(A,B)** The number of identified DP phosphopeptides and phosphoproteins shown by Venn diagram in 0, 3, and 12 h. **(C)** Distribution of the DP phosphoproteins in subcellular compartments. **(D)** Hierarchical clustering analysis of the DP proteins among 0, 3, and 12 h.

Phosphorylation is a reversible, highly dynamic post-translational modification. Therefore, an altered phosphorylation pattern usually indicates the potential function of phosphorylation in the corresponding biological process. In this study, we collectively identified 3434 phosphopeptides which showed a DP pattern among the three time points of BR treatment (|log2(fold-change)|≥ 1, *P* < 0.05) (**Table 1** and Supplementary Table S1). Of these, 598 were phosphorylated at 0 h, 214 at 3 h and 219 at 12 h, while the equivalent numbers of non-phosphorylated peptides were 136, 151, and 589 (**Figure 3A**); the remaining 1,527 peptides were phosphorylated at the three time points, but to a significantly varied degree (*P* < 0.05). A search of the set of known rice proteins allowed the DP peptides to be mapped onto 1,821 proteins, among which, respectively, 260, 92, and 100 were phosphorylated at the three time points, and respectively, 70, 81, and 252 were non-phosphorylated (**Figure 3B**). Of potential interest was that about 10% (191/1,821) of the proteins were either kinases or phosphatases, enzymes which are involved in protein phosphorylation/dephosphorylation, suggesting that these proteins are potential components of the BR signaling cascade. And indeed, several known BR signaling-related kinases, including OsGSK1 and OsSERK1 were detected to be DP (Koh et al., 2007; Li et al., 2009; Park et al., 2011). Rice genome contains at least 555 epi-genetic controlling factors (Gendler et al., 2008). Hou et al. (2015) reported that HDT701 and 27 other epigenetic controlling factors were DP in response to the *Xoo* infection, from which the proposal was that phosphorylation switch overriding the epi-genetic regulation may be a very universal model in the plant disease resistance pathway. In consistence with the report, the BR treatment also significantly altered the phosphorylation of 54 epigenetic controlling factors (involved in DNA methylation, histone methylation, histone acetylation and chromatin remodeling) and 118 transcription factors belonging to the families such as bHLHs, bZIPs, C3Hs, and Mybs. An analysis based on the CELLO algorithm showed that 52, 15, 14, and 10% of the DP proteins were located in the nucleus, chloroplast, cytoplasm, and plasma membrane, respectively. In contrast, proteins in the remaining 5 compartments such as mitochondrial, ER, and golgi accounted only less than 10% in total (**Figure 3C**).

**Table 1:** Some of the functionally reported brassinosteroid (BR)-induced differentially phosphorylated (DP) proteins in this study.

Peptide sequence	Locus ID	Annotation	Abbreviation	0 h intensity	3 h intensity	12 h intensity	Reference
VLVPGEPNISyICSR	LOC_Os01g10840.1	CGMC_GSK.2	OsGSK1	7.45E+07	3.99E+07	1.80E+07	Koh et al., 2007
GVSRPPStPASK	LOC_Os01g33040.1	Kinesin motor domain containing protein	DBS1/OsNACK	0.00E+00	4.24E+07	2.87E+07	Sazuka et al., 2005
EVHAsGELR	LOC_Os01g64970.1	CAMK_CAMK_like.11	SAPK4/OSPDK	0.00E+00	0.00E+00	2.04E+07	Diedhiou et al., 2008
VHSsSADPFSTLVGESPQFPDLGR	LOC_Os02g08500.1	Two-component response regulator	ORR4	5.65E+07	2.53E+07	0.00E+00	Ito and Kurata, 2006
StVGTPAYIAPEVLSR	LOC_Os02g34600.1	Calcium/calmodulin depedent kinases	SAPK6	0.00E+00	0.00E+00	1.79E+07	Chae et al., 2007
SGsAGEPLLR	LOC_Os02g35190.2	chloride channel protein	OsCLC-2	1.20E+08	2.07E+07	0.00E+00	Nakamura et al., 2006
GLVPVGGGGGsGRHEAALK	LOC_Os02g35970.1	NPH3 domain containing protein	CPT1	4.42E+07	3.86E+07	0.00E+00	Haga et al., 2005
sQPAELSR	LOC_Os02g36974.1	14-3-3 protein	GID2	3.49E+08	2.47E+08	1.20E+08	Ueguchi-Tanaka et al., 2000; Gomi et al., 2004
QSHsDGSLDTMAR	LOC_Os02g50550.1	Dynamin	BC3/OsDRP2B	2.75E+07	2.43E+07	0.00E+00	Xiong et al., 2010
SsPHGGLDDQIER	LOC_Os03g59060	Phosphatase 2A isoform 2	OsPP2Ac-2	4.00E+08	3.27E+08	1.22E+08	Yu et al., 2005
YDsGEIGHPK	LOC_Os03g59340.1	Cellulose synthase	OsCESA2	1.59E+07	0.00E+00	0.00E+00	Tanaka et al., 2003
AANAGMVAGsR	LOC_Os05g08370.1	Cellulose synthase	OsCESA1	2.57E+07	5.49E+06	0.00E+00	Tanaka et al., 2003
YVIsPDNQEIGEK	LOC_Os05g26890.1	G-protein alpha subunit	D1/RGA1	1.82E+07	5.71E+06	9.44E+05	Wang et al., 2006
DNLQGSAFLGSsR	LOC_Os07g06130.2	Ethylene-insensitive protein	OsEIN2/MHZ7	0.00E+00	4.97E+06	0.00E+00	Jun et al., 2004; Ma et al., 2013
MDGDADAVKsGR	LOC_Os07g10770.1	Cellulose synthase	OsCESA8	2.19E+07	0.00E+00	0.00E+00	Tanaka et al., 2003
HPFFAVsAPAsPTR	LOC_Os07g39220.1	BES1/BZR1 homolog protein	OsBZR1	7.20E+07	1.77E+07	0.00E+00	He et al., 2002; Wang et al., 2002; Bai et al., 2007
ADSPNPSSGDHPAGVGGsPEK	LOC_Os07g39480.1	WRKY and zinc finger domains	OsWRKY78	3.04E+07	1.19E+07	0.00E+00	Zhang et al., 2011
IHPYPVSEPGsAK	LOC_Os09g25490.1	Cellulose synthase	OsCesA9	0.00E+00	1.05E+07	0.00E+00	Tanaka et al., 2003
MEEsVGSR	LOC_Os10g27050	Phosphatase 2A isoform 4	OsPP2Ac-4	1.96E+07	3.58E+07	0.00E+00	Yu et al., 2005
MDTAsVTGGEHK	LOC_Os10g32980.1	Cellulose synthase	OsCesA7	7.42E+07	1.29E+07	6.26E+06	Tanaka et al., 2003


*The lower cases in peptide sequence indicate the phosphorylation sites.*

Based on the phosphorylation intensity, a hierarchical clustering analysis divided the DP proteins into seven groups (**Figure 3D**). The various phosphorylation dynamic tendencies suggested different roles of the DP proteins in each group. For example, the members of group V were most strongly phosphorylated at 3 h, and much less so at 0 and 12 h, indicating their involvement in the early response to BR. Those in group VII, in contrast, may be involved in the later response, because their level of phosphorylation remained low throughout the sampling period. To our surprise, the majority of DP proteins belonged to group I, in which the level of phosphorylation declined over time.

### Protein–Protein Interaction (PPI) Network of DP Proteins

**FIGURE 4:**
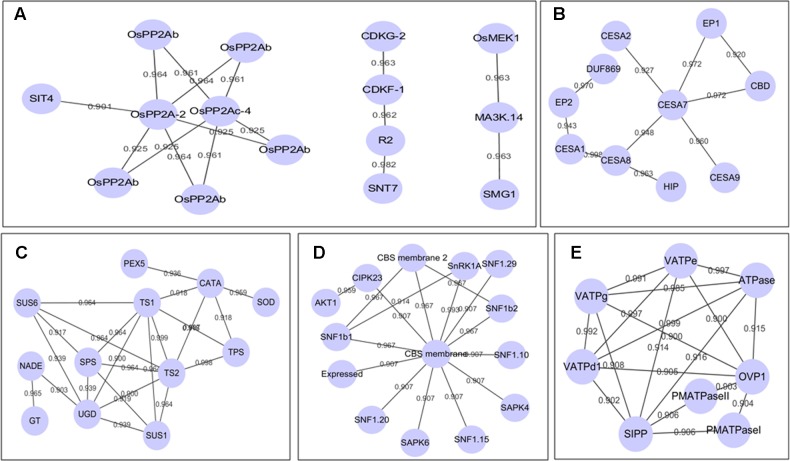
**(A–E)** Sub-network of all the DP proteins by using STRING and Cytoscape. The locus ID of the abbreviations in **(A–E)** could be seen in additional Supplementary Table S2.

By using the String 10.0^5^ (Search Tool for the Retrieval of Interacting Genes/Proteins) (Szklarczyk et al., 2015) and Cytoscape visualization (Shannon et al., 2003), we constructed PPI networks for DP kinases/phosphatases and all the BR induced DP proteins, respectively. **Figure 4A** depicted the sub-networks of DP kinases/phosphatases with high confidence score of 0.9. Eight PP2A phosphatases formed 11 edges (interaction relationships) sub-network, of which PP2A-2 (LOC_Os03g59060) and PP2Ac-4 (LOC_Os10g27050) were in the center. In *Arabidopsis* BR signaling, PP2As are critical phosphatases to dephosphorylate BZR1 and BZR2, which helps to retain the functional BZR1 and BZR2 in the nucleus and confer plants with BR response. The DP pattern suggested critical roles of these PP2As in BR response, whereas the predicted interaction relationships implied that PP2As may work in forms of protein complexes in BR signaling. A CDKG-2-CDKF-1-R2-SNT7 pathway was revealed in our PPI analysis. CDKG-2 (LOC_Os04g41100), CDKF-1 (LOC_Os06g22820), and R2 (LOC_Os05g32600) are cycling-dependent kinases functioning in cell proliferations, indicating this to be a key pathway in BR-regulated cell proliferation. In addition, we also detected a putative OsMEK1-MA3K.14-SMG1 pathway which is related to rice cell proliferation as well as stress resistance (**Figure 4A**). The full set of 1,821 DP proteins produced an interactome map composed of 459 nodes (proteins) and 946 edges. The various PPI sub-networks are associated with cellulose biosynthesis (**Figure 4B**), oxidation-reduction reactions (**Figure 4C**), phytohormone signaling (**Figure 4D**), and vascular ATP synthesis/transportation (**Figure 4E**).

### Validation of the Phosphorylation Pattern of DP Proteins

**FIGURE 5:**
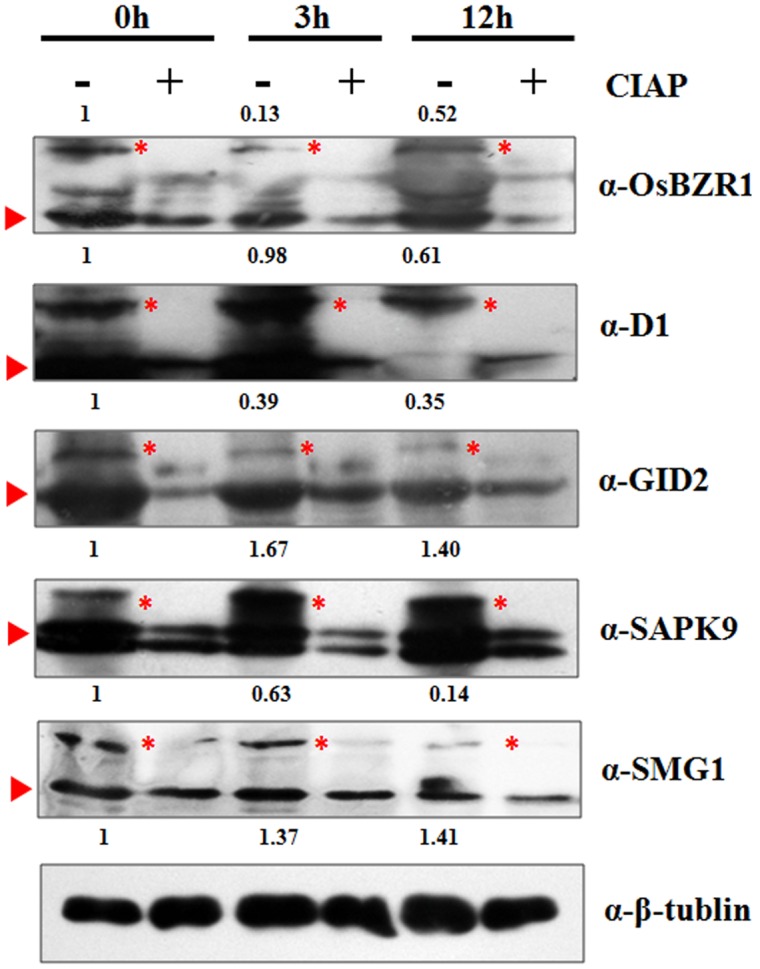
**Western-blot analysis of five selected proteins to verify the MS identified phosphorylation pattern at 0, 3, and 12 h.** Red triangle indicates the target band in original size; Red asterisk indicates the phosphorylated target protein band. Anti-tubulin was used as an internal control for normalization. The values above the phosphorylated bands represent the normalized, relative band intensities by setting 0 h into 1.

To validate the MS identified phosphorylation status, we performed Western-blot analysis for seven selected DP protein in the time course of BR treatment, including BZR1 (LOC_Os07g39220), D1 (LOC_Os05g26890), GID2 (LOC_Os02g36974), SAPK9 (LOC_Os12g39630), and SMG1 (LOC_Os02g54600). Due to the phosphates attachment, phosphorylated protein bands migrate more slowly through the gel than the unmodified proteins. By using β-tubulin as an internal control, the intensity of each phosphorylated band was semi-quantified. Fit well with our expectations, all the five DP proteins showed similar phosphorylation tendencies as the phosphoproteomic data indicated, though the extent of phosphorylation may vary from each other (**Figure 5**). The reduced phosphorylation status of BZR1, D1, GID2, and SMG1 in response to the BR treatment was confirmed by the Western blot result, as was the induced increase in the level of phosphorylation for SAPK9. Moreover, the absence of the slower-migrating bands following treatment with CIAP (Calf Intestine Alkaline Phosphatase) confirmed their identity as phosphorylated proteins. The results above strongly suggested that our phosphoproteomic data is highly reliable and such a MS-based quantification strategy could be applied for the phosphorylation dynamic detections in other biological processes.

## Discussion

After decades of research, the impact of BR is increasingly being recognized. The signaling of BR in plants largely relies on the transfer of phosphate groups among the signaling cascade members. As the first step toward understanding the mechanism of BR signaling, profiling the BR-induced phosphosites, phosphoproteins and phosphorylation dynamics is certainly fundamental and crucial. In the current study, a set of over 4,000 phosphosites related to nearly 2,000 rice phosphoproteins has been described. Given that the BR applied for treatment was dissolved in ethanol, there are concerns about the effects of solvent on the phosphoproteomic changes, even though the ethanol solvent has been diluted to 0.05% (v/v) (20 mM brassinnolide stock in 100% ethanol diluted to 10 μM in water). To verify this, we examined the transcriptional levels of three BR-responsive genes in response to 0.05% ethanol treatment by qRT-PCR. As shown in **Supplementary Figures S1D**–**F**, 0.05% ethanol treatment did not alter the transcriptional level of the three genes, indicating the solvent imposed ignorable effects on the rice seedlings. Most of these proteins were DP at some stage during the BR treatment, and a functional analysis suggested that many of them are involved in BR and other phytohormone signaling.

### Differential Phosphorylation on the Core Components of BR Signaling

According to the constructed BR signaling pathway in *A. thaliana*, the initial sensing of BR involves the cell membrane receptor BRI1, from which the signal is passed through the phosphorylation-mediated cascade BSK1-BSU1-BIN2/GSK1, finally reaching the transcription factor BZR1 which triggers the BR response (Wang et al., 2012; Wang W. et al., 2014). The present phosphoproteomic analysis revealed that both of the BR signaling core components OsGSK1 (Glycogen Synthesase Kinase3-like 1) (LOC_Os01g10840) and OsBZR1 (LOC_Os07g39220) were DP. OsGSK1 belongs to the plant GSK3/SHAGGY-like protein kinase family, and is a close ortholog of AtBIN2. Meanwhile, *OsBZR1*, encoding a transcription factor with a DUF822 domain, shared extensive sequence homology with *AtBZR1*. The ectopical expression of OsGSK1 led to a stunted plant growth, which mimicked the typical phenotype of the BR-deficient mutants. Therefore, OsGSK1 might serve as a negative regulator of BR signaling, just as its ortholog BIN2 does in *A. thaliana* (Koh et al., 2007). The function of BZR1 also appears to be conserved between *A. thaliana* and rice. *OsBZR1* RNAi suppressing lines showed dwarfism, erect leaves and reduced BR sensitivity, which is almost identical to the *bzr1-D* in *A. thaliana* (Wang et al., 2002; He et al., 2005).

A very interesting cytoplasm-nucleus shuttling model has been proposed for the BR signaling in the step from BIN2 to BZR1. In absence or low level of BR, upstream components of the cascade phosphorylate BIN2 to activate its kinase activity, which in turn catalyzes the phosphorylation on BZR1. The phosphorylation on BZR1 promotes its binding with a 14-3-3 protein, which allows the transport of BZR1 from the nucleus to the cytoplasm, where OsBZR1 remains non-functional. However, when the supply of BR is adequate, BIN2 is dephosphorylated, thereby losing its kinase activity; meanwhile, a PP2A de-phosphorylates BZR1, releasing it from its complex with the 14-3-3 protein, and allowing it to remain in the nucleus (Gampala et al., 2007). Bai et al. (2007) validated the 14-3-3 protein-mediated cytoplasm-nucleus shuttling mechanism of OsBZR1 in rice. Despite that they emphasized the binding of 14-3-3 to OsBZR1 retained its subcellular localization in cytoplasm under low BR level, the mechanistic basis of this binding has not be elucidated. The present experiment has shown that the extent of the phosphorylation affecting OsGSK1 and OsBZR1 was gradually reduced as a response to the BR treatment, in accordance with the behavior of BIN2 and BZR1 in *A. thaliana*. Perhaps more importantly, the differential phosphorylation of OsBZR1 implies that phosphorylation/dephosphorylation is likely to represent the means to determine whether it binds with 14-3-3 (as is the case in *A. thaliana*), which would mean that the BR signal transduction pathway is highly conserved between mono- and dicotyledonous plants. It is noteworthy that we failed to detect any phosphorylation on most of the reported BR signaling core components such as BRI1, BAK1, BSK1 and BSU1. In fact, a phosphoproteomic work of the BR signaling in *A. thaliana* encountered the same dilemma (Lin et al., 2015). The neglect of these proteins in the phosphoproteomic identification may be an artifact of low protein abundance and/or inadequate detection sensitivity. To overcome this, phosphorpoteomic works on more time points during the BR induction or different rice tissues might be necessary for wider proteomic coverage.

### BR Alteres the Phosphorylation of Other Phytohormone Signaling Proteins

Plant growth and development is fine-tuned by profound cross-talk between the various phytohormones. The interaction between BR and other hormones has been extensively studied (Nemhauser et al., 2006; Vanstraelen and Benkova, 2012). Though GA and BR both are growth-promoting hormones, research in *A. thaliana* revealed an antagonistic relationship between them (Bouquin et al., 2001). In the rice root, exogenously supplied BR simultaneously represses certain GA synthesis genes, while promoting GA homeostasis genes, thereby finally reducing the level of bioactive GA present (De Vleesschauwer et al., 2012). In addition, the interaction of the GA and BR signaling pathway may also contribute to the complexity of their cross-talk. Some BR-insensitive mutants were found to be compromised in sensing GA signal, while some GA-hypersensitive mutants even showed enhanced sensitivities to BR (Gallego-Bartolome et al., 2012). Here, the two GA signaling proteins GID2 (LOC_Os02g36974) and D1/RGA1 (LOC_Os05g26890) were both down-phosphorylated in response to the BR treatment. *gid2* and *d1/rga1* exhibited GA-insensitivity and typical GA-defective phenotype such as dwarf, erected panicles, indicating that both of them are positive regulators of GA signaling (Ueguchi-Tanaka et al., 2000; Gomi et al., 2004). Through a SCF^GID2^-proteasome pathway, GID2 mediates the degradation of GA signaling repressor SLR1 to activate the GA signal transduction (Gomi et al., 2004). D1/RGA1 is involved in GA and BR signaling, and it participates in the GA signaling majorly *via* a G protein-dependent pathway (Wang et al., 2006). *SLR* and *GID2* act epistatic over *D1* (Ueguchi-Tanaka et al., 2000). As we mentioned above, previous studies focused to explain the BR-GA antagonism from the view of quantity changes of GA synthesis and/or signaling genes in the transcriptional/translational levels. Nevertheless, the BR-induced down-phosphorylation of GID2 and D1/RGA1 is suggestive of a novel mechanism whereby BR inhibits the GA response by shutting down GA signaling. In addition to the GA signaling components, we also found that OSRK1/SAPK6 and SAPK4 involving in the ABA signaling (Chae et al., 2007; Diedhiou et al., 2008), CPT1 participating in auxin response (Haga et al., 2005) and OsEIN2 related to ethylene signaling (Jun et al., 2004) were DP in this study, suggesting a critical role of protein phosphorylation in the interplay between BR and other phytohormones.

### DP Proteins Related to Rice Architecture

One of the most direct effects of BR on plant is the altering of plant architecture, including plant height, leaf angle and tiller numbers (Zhang C. et al., 2014). Though BR defective mutants usually display unfavorable agronomic traits like dwarf, decreased leaf angle and more tillers, proper manipulation of the BR related genes was regarded as an effective way to improve crop architecture and eventually increase the yield (Sakamoto et al., 2006). Therefore, sorting out the protein connecting BR and plant architecture establishment will help us to construct the regulatory network and provide more gene resources for genetic improvement. As we expected, several rice architectures controlling proteins were found in the DP protein list. Cellulose synthesis for cell wall is a major determinant for the building of plant architecture and mechanic strength. Cellulose is synthesized at the plasma membrane by a complex containing multiple CESAs (cellulose synthase catalytic subunits) in plants. In rice, at least 10 CESAs (OsCESA1-10) have been identified, the contribution of three of which (*OsCesA4, A7* and *A9*) has been defined by analysis of loss-of-function mutants, which all express a brittle leaf phenotype (Tanaka et al., 2003). Our PPI analysis of the DP proteins revealed a cellulose synthesis network containing five CESAs, which included OsCESA1, 2, 7, 8, and 9. The suggestion is therefore that BR influences cellulose synthesis and thereby has a profound effect on the architecture of the plant. Consistent with this notion, it was observed that several other cellulose synthesis-related proteins were DP. For example, BC3 (Brittle Culm3), a dynamin-related protein, is responsible for the membrane trafficking between the plasma membrane and intracellular compartments, which is an important process that regulates the deposition and metabolism of cellulose of the second cell wall. Mutation of *BC3* led to inferior mechanical properties in rice plants (Xiong et al., 2010). The BR-induced DP pattern of multiple CESAs and other cellulose-related proteins in the current study strongly implied that BR regulates rice architectures by controlling cellulose synthesis *via* a phosphorylation-dependent mechanism.

### DP Antioxidant Enzymes

Reactive oxygen species (ROS) production, including superoxide radical, hydroxyl radical and hydrogen peroxide (H_2_O_2_) is implicated as important regulatory and signaling elements for plants to adapt to unfavorable environments. It has been reported that application of exogenous BRs modified a wide range of antioxidant enzymes to counter the oxidative stress led by various environmental stimulus (Bajguz and Hayat, 2009). For example, application of BR promoted the activities of superoxide dismutase (SOD), catalase (CAT), ascorbate peroxidase (APX) in maize seedlings under water stress as well as in rice exposed to saline stress (Li et al., 1998; Núñez et al., 2003). Nevertheless, it is not clear yet that how BRs promote the antioxidant enzyme activities in response to stresses. Interestingly, we found that several DP antioxidant enzyme proteins such as CATA (LOC_Os02g02400), SOD (LOC_Os03g22810), and PEX5 (LOC_Os08g39080), which hinted that BR-induced protein phosphorylation may be involved in the adjustment of the activities of these antioxidant enzymes. Peroxisomes are single membrane-bound organelles where generation or degradation of ROS occurs. Peroxisomal proteins are originally translated in the cytoplasm and transported into peroxisomes. PEX5 is a PTS (Peroxisomal targeting signal) receptor protein, which recognizes the PTS and is responsible for the translocation of the peroxisomal proteins. In *A. thaniana*, *Atpex5* mutant loses germinability in the absence of sucrose, but could be rescued by PEX5. PEX5 was also able to translocate PTS-containing proteins into the peroxisome by interacting with OsPEX7p (Lee et al., 2006). SOD is a type of key enzymes in antioxidant defense, which catalyze the partitioning of the superoxide radical into oxygen or less harmful H_2_O_2_, thus to alleviate the cell damages caused by oxidative stresses. Catalases act as scavengers of H_2_O_2_, and could further decompose it into oxygen and water. In rice, it has been known that CATB functions in an ABA-dependent manner to prevent the excessive accumulation of H_2_O_2_ under water stress (Ye et al., 2011). CATs are also targets of excess copper toxicity, which leads to retarded seed germination in rice (Ye et al., 2014). A very recent study suggested that the physical association-dissociation of GLO (Glycolate Oxidase; H_2_O_2_ producer) and CAT serve as a specific machinery to modulate H_2_O_2_ levels in rice (Zhang et al., 2016). Moreover, a potential PEX5-CATA-SOD interaction relationship was suggested by our PPI analysis (**Figure 4C**). Hence, we proposed that exogenous BR alters the activity of antioxidant enzymes *via* changing the phosphorylation status and/or intensity on antioxidant enzymes, and finally confers plants with resistance to environmental stresses.

## Author Contributions

YH, JQ, YW, ZL, JZ, XT, and HL performed the experiments and analyzed the data, JZ conceived of the project, designed and coordinated the experiments, and wrote the manuscript. All the authors read and approved the final manuscript.

## Conflict of Interest Statement

The authors declare that the research was conducted in the absence of any commercial or financial relationships that could be construed as a potential conflict of interest.

## Footnotes

1 ftp://ftp.plantbiology.msu.edu/pub/data/Eukaryotic_Projects/o_sativa/annotation_dbs/pseudomolecules/version_7.0/all.dir/

2 http://rsb.info.nih.gov/ij/docs/menus/analyze.html#gels

3 http://motif-x.med.harvard.edu/

4 http://www.cbs.dtu.dk/services/NetSurfP/

5 http://string-db.org/

## Supplementary Material

The mass spectrometry proteomics data have been deposited to the ProteomeXchange Consortium *via* the PRIDE partner repository with the dataset identifier PXD004705.

**FIGURE S1 | qRT-PCR analysis of the time-course expression of genes in response to BR** **(A–C)** or 0.05% ethanol solvent treatment **(D–F)**. Ubiquitin gene was used as the internal control and error bars indicate the SD from three biological replicates. Significance of differences was determined by one-way ANOVA tests. Different characters indicate a statistically significant difference at *P* < 0.05.
