# Supplementary figures and images for: A Quantitative Proteomic Analysis of Brassinosteroid-induced Protein Phosphorylation in Rice (Oryza sativa L.)
Source: Front Plant Sci. 2017 Apr 7;8:514. doi: 10.3389/fpls.2017.00514 (PMC5383725; doi:10.3389/fpls.2017.00514)

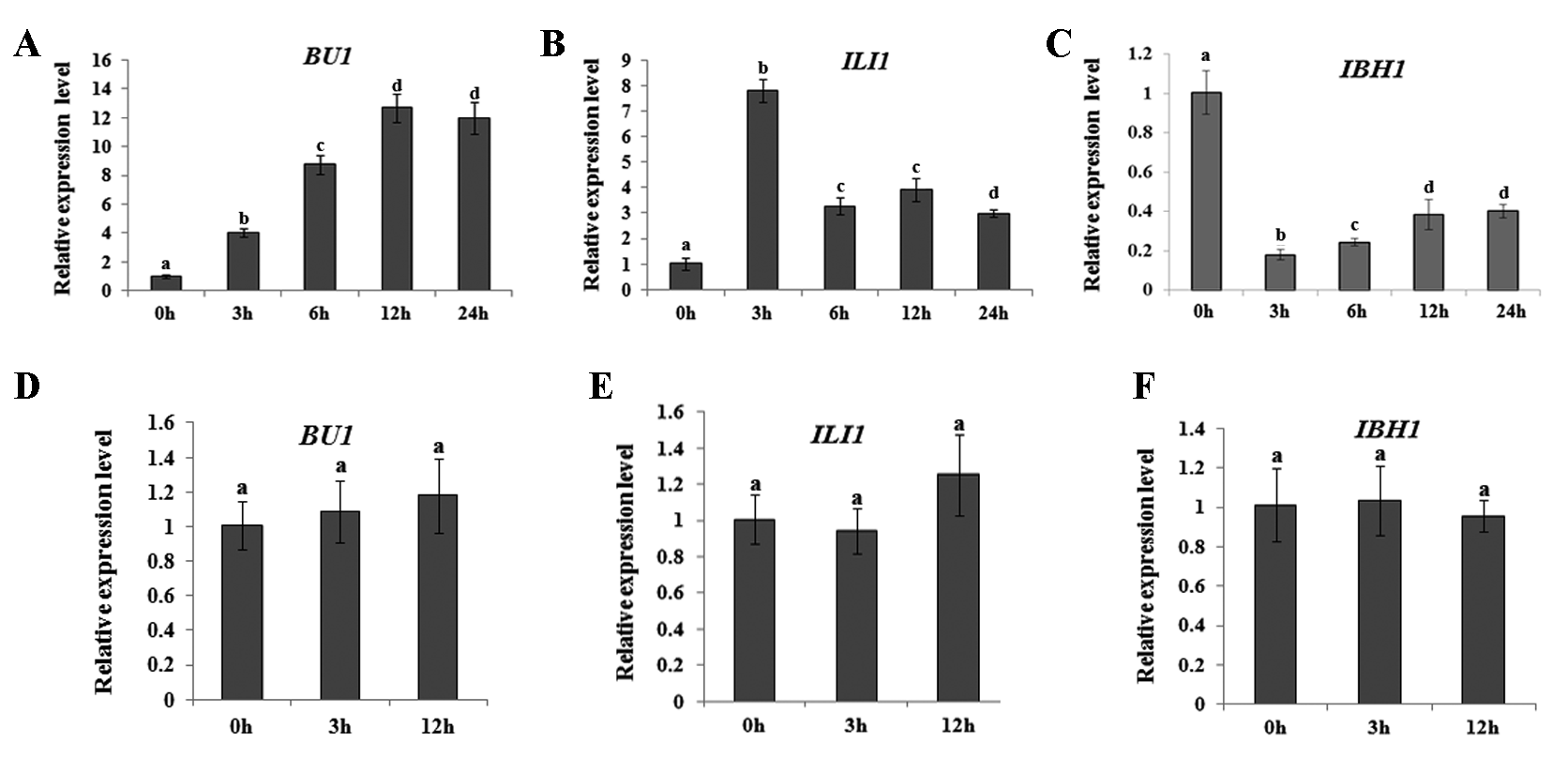

Supplement: Supplementary file 1 [file Image_1.TIF]
